# Human skin aging is associated with increased expression of the histone variant H2A.J in the epidermis

**DOI:** 10.1038/s41514-021-00060-z

**Published:** 2021-04-01

**Authors:** Claudia E. Rübe, Caroline Bäumert, Nadine Schuler, Anna Isermann, Zoé Schmal, Matthias Glanemann, Carl Mann, Harry Scherthan

**Affiliations:** 1 Saarland University Hospital, Department of Radiation Oncology, Homburg/Saar, Germany; 2 Saarland University Hospital, Department of Visceral Surgery, Homburg/Saar, Germany; 3 Université Paris-Saclay, CEA, CNRS, Institute for Integrative Biology of the Cell (I2BC), Gif-sur-Yvette Cedex, France; 4 Bundeswehr Inst. of Radiobiology affiliated to the Univ. of Ulm, München, Germany

**Keywords:** Epigenetics, Ageing

## Abstract

Cellular senescence is an irreversible growth arrest that occurs as a result of damaging stimuli, including DNA damage and/or telomere shortening. Here, we investigate histone variant H2A.J as a new biomarker to detect senescent cells during human skin aging. Skin biopsies from healthy volunteers of different ages (18–90 years) were analyzed for H2A.J expression and other parameters involved in triggering and/or maintaining cellular senescence. In the epidermis, the proportions of H2A.J-expressing keratinocytes increased from ≈20% in young to ≈60% in aged skin. Inverse correlations between Ki67- and H2A.J staining in germinative layers may reflect that H2A.J-expressing cells having lost their capacity to divide. As cellular senescence is triggered by DNA-damage signals, persistent 53BP1-foci, telomere lengths, and telomere-associated damage foci were analyzed in epidermal keratinocytes. Only slight age-related telomere attrition and few persistent nuclear 53BP1-foci, occasionally colocalizing with telomeres, suggest that unprotected telomeres are not a significant cause of senescence during skin aging. Quantification of integrin-α6+ basal cells suggests that the number and function of stem/progenitor cells decreased during aging and their altered proliferation capacities resulted in diminished tissue renewal with epidermal thinning. Collectively, our findings suggest that H2A.J is a sensitive marker of epidermal aging in human skin.

## Introduction

Cellular senescence may contribute to the physiological aging process in human tissues^1,2^. Senescence typically occurs in response to damaging stimuli, such as telomere shortening or dysfunction (replicative senescence) or persistent activation of the DNA-damage response (DDR)^3^. Senescent cells are characterized by stable cell cycle arrest, morphological and metabolic changes, chromatin reorganization, and altered gene expression. The adoption of a pro-inflammatory phenotype known as the senescence-associated secretory phenotype (SASP) may promote tissue inflammation and deterioration^4,5^. Growing evidence suggests that senescent cells accumulate in tissues and organs with advancing age, thereby impairing physiological regeneration processes and contributing to organismal aging^6^.

The biological role of cellular senescence in human skin aging is still largely unknown^7^. The skin covers the entire external surface of the human body and protects against major environmental stresses. The human epidermis consists primarily of keratinocytes and corneocytes, continually formed through keratinocyte differentiation, and is stratified in the following cell layers: the stratum basale, the deepest layer, consists of cuboidal basal cells attached to the basal lamina and comprises epidermal stem/progenitor cells. Basal cells and the deepest cells within the stratum spinosum undergo mitosis, forming new keratinocytes that move into more superficial layers and complete their natural maturation program. By the time keratinocytes reach the stratum granulosum, they flatten and produce more keratin filaments and lipid-filled membrane-coated vesicles, thereby forming a water-resistant protective barrier. The stratum corneum consists of numerous layers of flattened, dead corneocytes lacking organelles and nuclei. During their constant differentiation, human keratinocytes migrate in 30–40 days from basal to cornified layers, and finally desquamate from the skin surface. Epidermal homeostasis depends on the regenerative capacity of stem/progenitor cells and on complex signaling pathways that orchestrate tissue morphogenesis^8^.

Accumulating evidence indicates that telomere erosion and/or dysfunction may induce replicative senescence and thus may contribute to the aging process^9^. Human telomeres are composed of tandem repeats of non-coding 5′-(TTAGGG)_n_-3′ sequences associated with the shelterin complex that facilitates the formation of t-loop structures to shield chromosomal termini from erosion and fusion with other chromosome ends^10,11^. The progressive loss of telomeric DNA occurs in all dividing normal cells owing to incomplete lagging-strand DNA synthesis. Telomere shortening eventually results in de-protection of chromosome ends triggering the DDR. Histone γH2AX and 53BP1 are sensitive markers of double-stranded DNA breaks (DSBs)^12–17^ and are engaged in DSB repair processes at de-protected telomeres^18^. Previous studies have shown that the number of DNA-damage foci increases in senescent cells in most tissues during aging^15,19^.

Growing evidence indicates that the physiological aging process is characterized by enormous changes in the chromatin organization. The conformation and dynamic properties of chromatin determine the access to the DNA sequence information and thus control many activities of the cell^20–23^. Particularly, the replacement of canonical histones by histone variants can lead to profound modifications of the chromatin structure^24^. Previous studies have shown that histone variant H2A.J is expressed and incorporated into the chromatin of human fibroblasts during replicative senescence, which in turn modifies chromatin properties and induces the expression of inflammatory genes^25^. Histone variant H2A.J is expressed replication-independent and differs from canonical H2A by one amino-acid exchange (Val-11 instead of Ala-11) and the C terminus containing an SQ motif. Recent work elucidates the functional significance of H2A.J-specific residues and proposes potential mechanisms in promoting the SASP^26^. Accordingly, H2A.J incorporation mitigates the binding of histone H1 to the nucleosome and thereby increases the dynamic H1 exchange on chromatin. The decreased association between H1 and chromatin leads to the expression of STAT/IRF transcription factors with transcriptional activation of interferon-stimulated genes^26^. Moreover, our recent immunoelectron microscopy studies of human fibroblasts exposed to high doses of ionizing radiation (IR) revealed that H2A.J is deposited into persistent DNA damage during the progression of radiation-induced senescence^27^.

There is some evidence that the accumulation of senescent cells with advancing age may contribute to age-related changes of the skin^28^. However, the discovery of reliable markers that enables the detection of senescent cells in human skin still represents a major challenge to study the interrelation between cellular senescence and aging^4^. Here, we investigated histone variant H2A.J as a potential biomarker to identify and enumerate senescent cells in the epidermis during human skin aging.

## Results

### Age-dependent accumulation of H2A.J in human epidermis

**Fig. 1:**
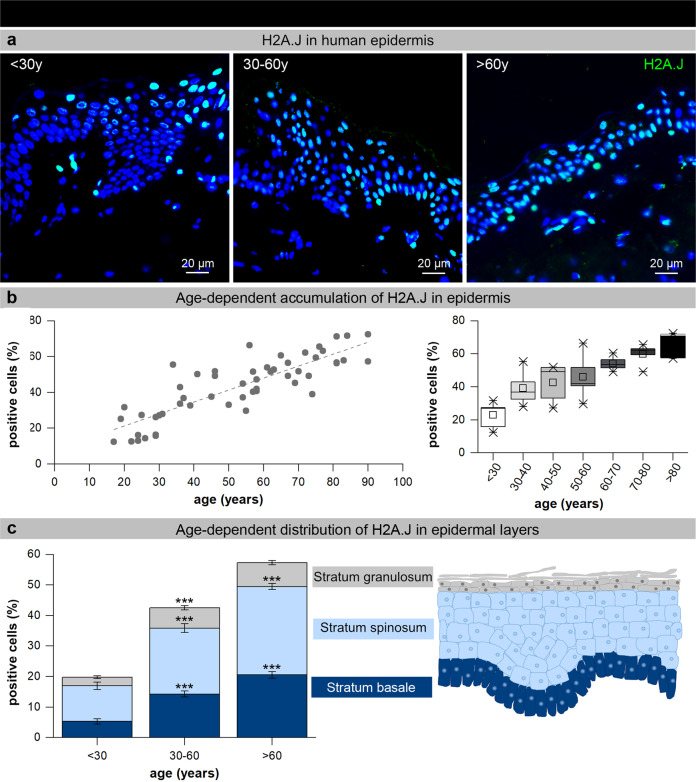
Age-dependent accumulation of H2A.J in human epidermis. **a** IF micrographs of H2A.J staining in human epidermis of young (<30 y), middle-aged (30–60 y), and aged (>60 y) donors. **b** Graphic presentation of H2A.J expression (H2A.J+ cells in percentages) depicted for the entire age cohort (*n* = 53; left panel) and depicted per age-group (right panel: the horizontal line in the box represents the median, the box shows the interquartile range [25th–75th percentile] and the whiskers represent the 5th–95th percentile.) **c** Graphic presentation of H2A.J expression (H2A.J+ cells in percentages) in different epidermal layers in relation to donor age. ***p < 0.001 significant statistical difference to younger age-group.

To test histone variant H2A.J as a novel biomarker for keratinocyte senescence, human skin samples were collected from volunteers of varying ages (18–90 years). Excisional biopsies from photo-protected skin were stained for H2A.J as well as established senescence markers to analyze the chronological appearance of cellular senescence. With increasing donor age, we observed pronounced accumulation of H2A.J+ keratinocytes in the epidermis (Fig. 1a). The percentage of H2A.J+ keratinocytes increased from ≈20% in young (<30 y) to ≈60% in aged skin (>60 y) (Fig. 1b, left panel). Although young (<30 y) and aged skin (>60 y) can be clearly distinguished based on their different H2A.J expression in the epidermis, skin samples of intermediate age groups (30–40 y, 40–50 y, 50–60 y) exhibited varying H2A.J levels (Fig. 1b, right panel), suggesting that other parameters than chronological age may influence H2A.J expression. Subsequently, relative proportions of H2A.J+ keratinocytes were analyzed in different epidermal layers (Fig. 1c). Stratum granulosum is the most superficial layer of the epidermis in which all keratinocytes still possess nuclei. Independently of donor age, we observed significant proportions of H2A.J+ keratinocytes (≈3–8%) in the granular layer of epidermis (Fig. 1c). During their final differentiation, epidermal keratinocytes are transformed from living cells into corneocytes, characterized by the loss of their nuclei. H2A.J may have physiological functions in this maturation process associated with cornification, or their presence may reflect the upward migration of H2A.J+ cells from lower epidermal layers. The deepest cells of the stratum spinosum remain capable of mitosis, but as they are pushed farther upward, they cease dividing. The age-dependent increase of spinous H2A.J+ keratinocytes from ≈11% in young (<30 y) to ≈28% in aged epidermis (>60 y) may reflect the age-associated decline of proliferative capacity, leading to the diminished epidermal thickness (Fig. 1c). Highly proliferative epidermal stem/progenitor cells dominate the basal layer. Our results revealed an age-dependent increase of H2A.J+ cells in basal layers, rising from ≈5% in young (<30 y) to ≈20% in aged epidermis (>60 y) (Fig. 1c). This age-dependent H2A.J expression in germinative layers may indicate the age-associated loss of regenerative capacity.

Validating an antibody’s specificity is crucial to ensuring the absence of non-specific binding. Owing to structural similarities between H2A.J, H2A, and H2A.X, we aimed to exclude potential cross-reactivity of our antibody, which would invalidate our results. However, double-staining for H2A.J with canonical H2A and histone variant H2A.X was technically not feasible, probably because their antigens are located on identical nucleosomal structures, impacting antibody binding by mutual steric hindrance. However, the single-staining patterns for canonical H2A and histone variant H2A.X in young, old, and irradiated human epidermis are distinctly different from H2A.J, with no visible variations due to age or radiation exposure (Supplementary Figure 1a). As expected, we observed only single γH2A.X-foci in non-irradiated epidermis of middle-aged and aged individuals, but multiple γH2A.X-foci in ex vivo irradiated human skin (10 Gy, 24 h post-IR) (Supplementary Figure 1a). Subsequently, the specificity of H2A.J staining in human epidermis was analyzed by combining the H2A.J antibody with peptides synthesized for the specific binding epitopes of H2A.J and H2A, respectively. In combination with H2A.J peptide, H2A.J staining in human epidermis was completely inhibited, but in combination with H2A peptide, we observed the age-dependent H2A.J staining pattern (Supplementary Figure 1b). These competition assays using the immunizing peptide to test for specificity, demonstrate that the H2A.J antibody binds specifically to the antigen against which it was raised. Testing antibody performance against genetically modified samples is another way to verify that an antibody recognizes a specific target. Accordingly, we analyzed murine epidermis of wild-type (WT) and H2A.J knock-out (KO) mice in relation to their age and exposed to IR, respectively. Although young WT mice (3 months old) revealed nearly no H2A.J staining, older (12 months old) and irradiated WT mice (2 Gy, 1w post-IR) revealed clearly higher H2A.J staining levels in their epidermis. In contrast, murine epidermis of KO mice (that do not express the target protein H2A.J) revealed absolutely no positive staining with the H2A.J antibody (Supplementary Figure 1c). Collectively, these control experiments provide definitive proof for specificity of our H2A.J antibody.

Multiple markers are generally mandatory for reliable identification of senescent cells within complex tissues. Senescence‐associated β‐Galactosidase (SA‐β‐Gal) is the most widely used biomarker, but unexpectedly our SA‐β‐Gal staining (performed on fresh skin samples under controlled pH conditions) did not accurately detect SA-β-Gal+ cells (Supplementary Figure 2). Analyzing skin biopsies of differently aged donors with other senescence markers, we observed p16^Ink4a^ expression and lipofuscin accumulation in basal layers of aged epidermis (Supplementary Figure 2).

### Proliferation and persistent DNA-damage foci during human skin aging

**Fig. 2:**
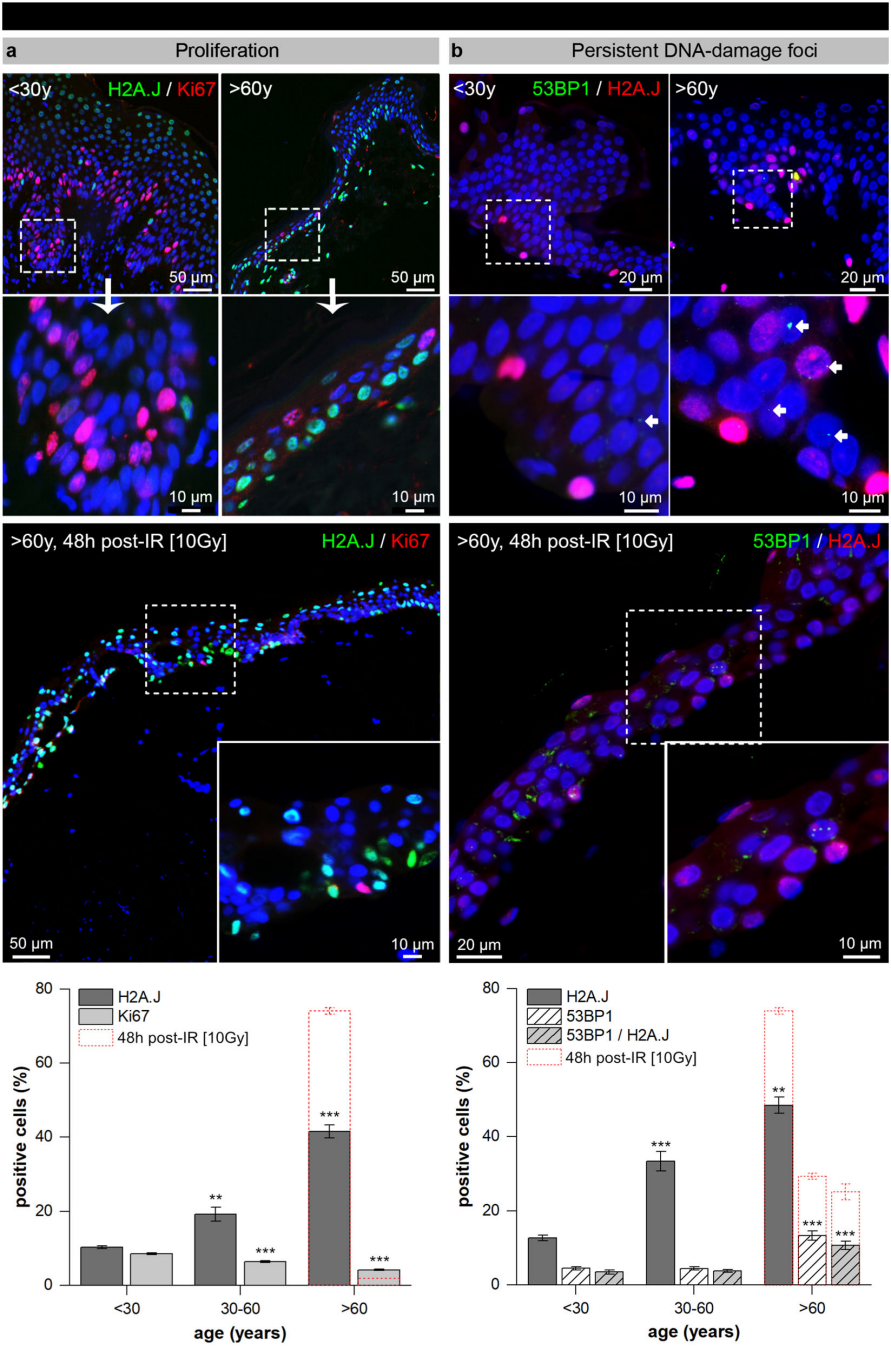
Proliferative capacity, DNA-damage foci, and H2A.J during human skin aging. **a** IF micrographs of H2A.J and Ki67 double-staining in young, aged, and irradiated skin (upper panel). Graphic presentation of the quantification of H2A.J+ and Ki67+ cells in epidermis of young (<30 y), middle-aged (30–60 y), and aged (>60 y) donors (*n* = 3 per age-group). **b** IF micrographs of 53BP1 and H2A.J double-staining in young, aged and irradiated skin. Graphic presentation of the quantification of 53BP1+, H2A.J+, and double-positive cells in epidermis of young (<30 y), middle-aged (30–60 y), and aged (>60 y) donors (*n* = 3 per age-group). Data are presented as mean±SE; **p < 0.01, ***p < 0.001 significant statistical difference to younger age-group. Red, broken lines in the bar graph indicate the results for irradiated skin.

Using Ki67 as a proliferation marker, we examined the regenerative capacity in human epidermis in relation to donor’s chronological age (Fig. 2a). The numbers of Ki67+ and H2A.J+ keratinocytes were quantified in identical areas (same cells) of double-stained skin sections. Our results show that Ki67+ cells were most abundant in skin biopsies of young donors (<30 y: 8.5 ± 0.3%). With increasing donor-age epidermal keratinocytes lost their proliferative capacity, and Ki67+ fractions decreased to ≈6% for middle-aged (30–60 y: 6.3 ± 0.3%) and to ≈4% for aged donors (>60 y: 4.2 ± 0.2%) (Fig. 2a). Moreover, using Ki67 in direct combination with H2A.J, we observed an inverse correlation between Ki67+ and H2A.J+ cells in every age-group; even only weakly H2A.J+ cells show no Ki67 expression (Fig. 2a). The fact that Ki67-H2A.J immunostaining was mutually exclusive, strongly suggests that cells expressing H2A.J have lost their ability to proliferate, as predicted if these cells are senescent.

Persistent DNA damage as a critical trigger of cellular senescence can be identified by DNA damage foci, such as γH2AX- and/or 53BP1-foci^12–14,16^. Double-staining for 53BP1 and H2A.J was used to quantify DNA damage foci and to detect senescent cells concurrently in the epidermis of human skin (Fig. 2b). Skin biopsies of young donors (<30 y) revealed only low percentages of 53BP1+ (4.4 ± 0.5%) and H2A.J+ (12.6 ± 0.7%) cells, as well as double-positive keratinocytes (3.4 ± 0.5%). In middle-aged donors the fraction of H2A.J+ keratinocytes increased significantly to ≈30% (30–60 y: 33.4 ± 2.7%), whereas the fraction of 53BP1+ and H2A.J+/53BP1+ cells remained at low levels. In the epidermis of aged skin, H2A.J+ keratinocytes increased to ≈40% (>60 y: 48.5 ± 2.2%) (Fig. 2b). However, in aged skin only ≈10% of H2A.J+ keratinocytes displayed persistent 53BP1-foci, suggesting that age-dependent accumulation of DNA damage is not the most important cause of cellular senescence.

To explore the role of DNA damage for induction of cellular senescence, cultured skin samples of aged donors were exposed to ionizing irradiation (IR, 10 Gy) and analyzed 72 h after ex vivo exposure. Analysis of this irradiated epidermis revealed significantly increased 53BP1-foci levels (53BP1+ cells: 29.4 ± 0.8%), likely reflecting un- or misrepaired DNA damage (Fig. 2b). The irradiated epidermis showed clearly reduced levels of Ki67+ keratinocytes (1.8 ± 0.1%) that could be explained by inhibition of proliferation owing to activation of DNA-damage checkpoints (Fig. 2a). Significantly, the percentage of H2A.J+ keratinocytes in this irradiated epidermis was clearly elevated (74.1 ± 0.9%), compared to non-irradiated, age-matched controls (Fig. 2). These findings support the close interrelationship between persistent DNA damage, inducing long-lasting cell cycle arrest in damaged epidermal keratinocytes, which is associated with increased H2A.J expression in the human epidermis.

**Fig. 3:**
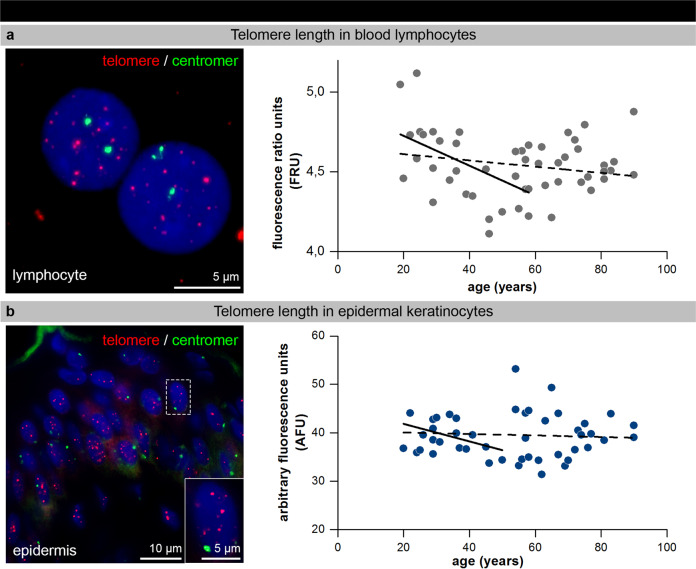
Telomere-length heterogeneity in blood lymphocytes and epidermal keratinocytes. **a** IF micrographs of telomere (hybridized with telomere-specific PNA probe, red) and centromere 2 (green) FISH staining in human blood lymphocytes (left panel). Graphic presentation of relative telomere-length measurements in blood lymphocytes of donors of varying age (*n* = 51) are given as fluorescence ratio units (FRU). Solid trend line indicates age-dependent relative telomere-length reduction up to 60 years. **b** IF micrographs of telomere (red) and centromere 2 (green) staining in human epidermal keratinocytes (left panel) of tissue sections. Graphic presentation of the mean relative telomere length as arbitrary fluorescence units per nucleus (AFU) in epidermal keratinocytes of donors of varying age (*n* = 51). Solid trend line indicates an age-dependent telomere-length reduction up to 50 years.

Telomere-length heterogeneity in blood lymphocytes and epidermal keratinocytes average telomere length, usually measured in human blood lymphocytes, is thought to be a biomarker for chronological aging. In our cohort of healthy individuals, relative telomere lengths were measured by interphase quantitative fluorescence in situ hybridization (IQ-FISH) in interphase nuclei of blood lymphocytes and epidermal keratinocytes (Fig. 3)^29^. Our results revealed remarkable inter-individual telomere-length heterogeneity, in both lymphocytes isolated from peripheral blood samples (Fig. 3a) and keratinocytes analyzed in epidermis sections (Fig. 3b), particularly among middle-aged donors (30–60 y). We observed only slight age-dependent telomere-length reductions until the age of 60 years in blood lymphocytes and until the age of 50 years in epidermal keratinocytes (solid lines in Fig. 3a, b). Since some older donors (>60 y) revealed similar mean telomere lengths as younger donors (<30 y), the overall trend of an age-dependent telomere attrition was attenuated (broken line in Fig. 3a, b). These findings may reflect the positive selection of healthy elderlies analyzed here that are physically fit for abdominal surgery. Our results suggest that intrinsic aging of human skin occurs rather independently of telomere erosion in epidermal keratinocytes^30^.

### Telomere-associated DNA damage foci in epidermal keratinocytes

**Fig. 4:**
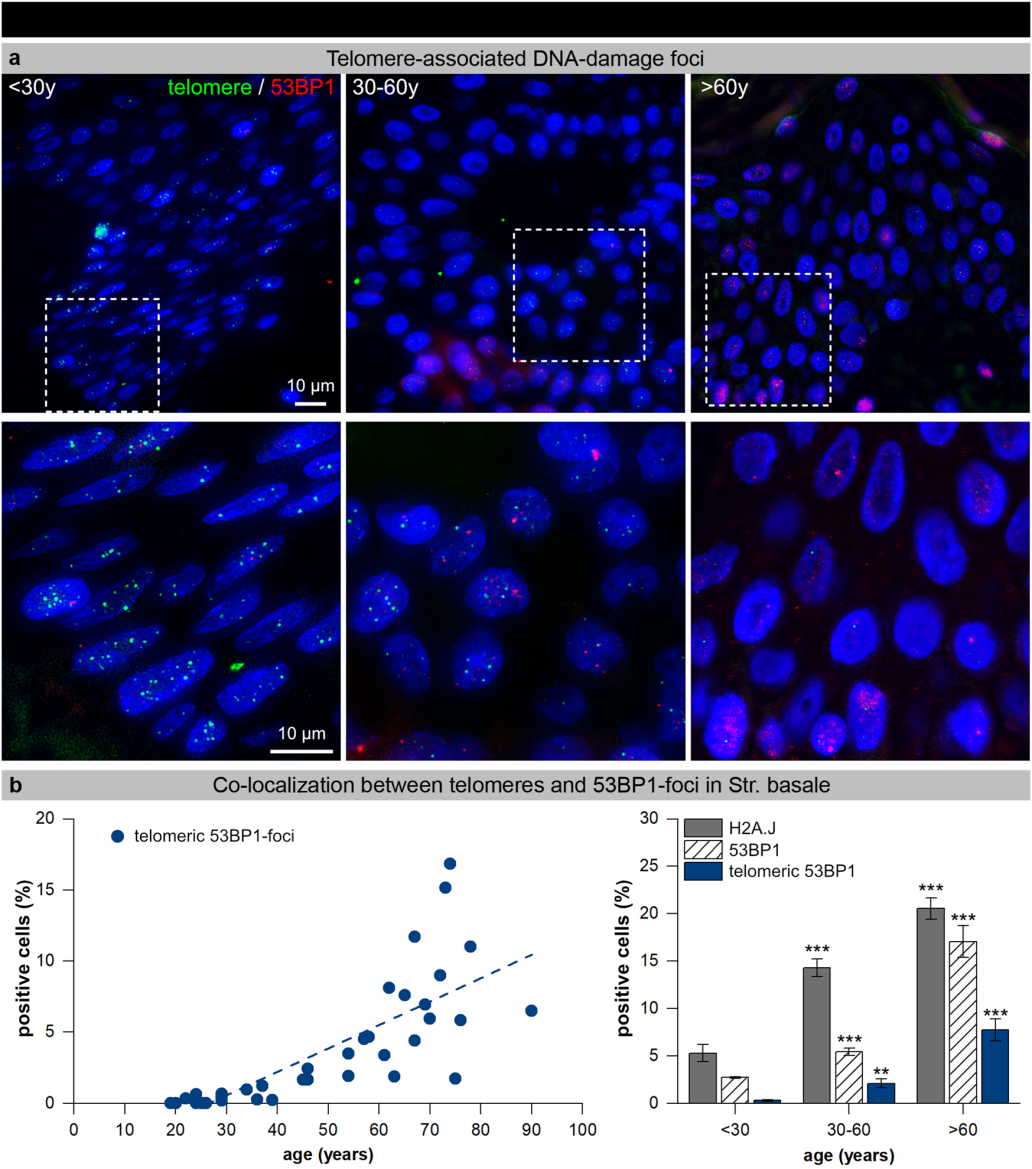
Telomere-associated DNA damage foci in epidermal keratinocytes. **a** Micrographs of telomere FISH signals (green) and 53BP1 IF signals (red) in human epidermal keratinocytes of young (<30 y), middle-aged (30–60 y) and aged (>60 y) skin. Selected areas are shown below at higher magnification. **b** Graphic presentation of epidermal keratinocytes quantification with telomeric 53BP1-foci in human skin of donors of varying age (left panel, *n* = 53). Graphic presentation of epidermal keratinocytes quantification with H2A.J expression, 53BP1-foci, and telomeric 53BP1-foci in the stratum basale of donors of varying age. Data are presented as mean±SE; **p < 0.01, ***p < 0.001 significant statistical difference to younger age-group.

As cellular senescence was previously shown to be induced by DDR signaling emanating from shortened telomeres, we next examined the occurrence of telomere-associated DNA damage foci in basal keratinocytes of human epidermis. Young and aged skin samples were analyzed by immunofluorescence microscopy (IFM) to detect the colocalization of DNA damage foci and telomeric DNA FISH signals in keratinocytes of the stratum basale (Fig. 4). In skin samples of young and healthy donors, cell nuclei of keratinocytes revealed clearly visible telomeric signals (Fig. 4a, left panel), but only few individual 53BP1-foci (<30 y: 2.8 ± 0.1%), and almost no telomere-associated 53BP1-foci (<30 y: 0.3 ± 0.1%) (Fig. 4b, right panel). In skin biopsies of aged donors, telomeric DNA sequences were less visible, likely due to their replicative attrition (Fig. 4a, right panel), but some nuclei displayed 53BP1 signals (>60 y: 17.0 ± 1.7%), of which only some colocalized with telomeric DNA (> 60 y: 7.7 ± 1.2%) (Fig. 4b, right panel). Collectively, our quantitative measurements revealed an age-dependent, but only slight increase of telomere-associated damage. However, the low incidence of telomere-associated 53BP1-foci suggests that temporal enrichment of dysfunctional telomeres is not a prominent cause for cellular senescence during human skin aging.

To analyze potential colocalizations between telomeres and 53BP1 at ultra-high resolution, we next performed immunogold-labeling to characterize telomere-associated DNA damage by transmission electron microscopy (TEM). In low-power electron micrographs, various epidermal layers are easily discernible and previous TEM studies have accurately characterized epidermal ultrastructures in young and aged human skin (Supplementary Figure 2a). In the keratinocyte nuclei of young epidermis, the detection of telomere-specific DNA sequences revealed gold-particle chains of considerable length, likely reflecting long telomere sizes at young age (Supplementary Figure 2b, left panel). In aged epidermis, by contrast, age-depending telomere attrition resulted in shorter chains of telomere-specific gold-beads, likely reflecting smaller telomere sizes at an advanced age (Supplementary Fig. 2b, right panel). Significantly, these telomere-specific sequences were always spatially distant from gold-labeled 53BP1, in both young and aged human skin, suggesting that there is no direct colocalization between chromosome ends and DDR factor 53BP1 at the nanoscale level (Supplementary Fig. 2b). However, critically short telomeres may fail to produce distinct in situ signals with our TEM techniques. Moreover, there remains the possibility that critically short telomeres may be signaled through 53BP1-independent mechanisms.

### Epidermal stem/progenitor cells and epidermis thickness during human skin aging

**Fig. 5:**
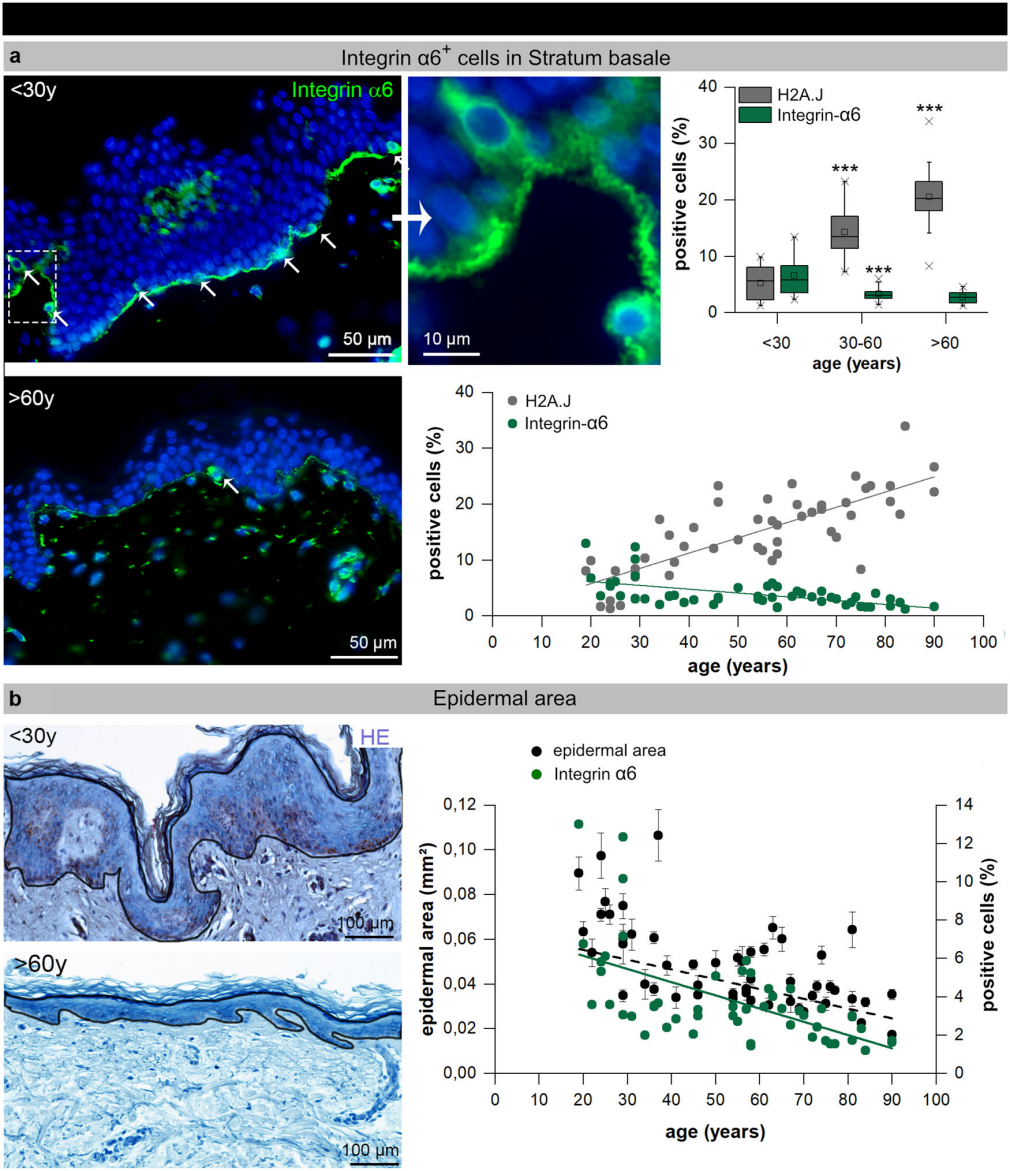
Epidermal stem cells and epidermal thickness during human skin aging. **a** IF micrographs of integrin-α6 expression in young and aged human skin. The selected area is shown at higher magnification (left panel). Quantification of integrin-α6+ cells in human epidermis of donors of varying age depicted per age-group (upper panel: The horizontal line in the box represents the median, the box shows the interquartile range [25–75th percentile] and the whiskers represent the 5–95th percentile) and for the entire age cohort (*n* = 52; lower panel). ***p < 0.001 significant statistical difference to younger age-group. **b** Micrographs of H&E stained human skin of young and aged donors. Measured areas of the epidermis (without corneal layer) are indicated by black lines. Graphic presentation of measured epidermal areas and integrin-α6+ cells in relation to donor-age (*n* = 52).

Previous studies suggest that stem and progenitor keratinocytes in human epidermis are characterized by high cell surface expression of integrin-α6^31^. To determine whether epidermal stem/progenitor cell abundance is altered during skin aging, integrin-α6+ basal cells were quantified by IFM (Fig. 5). IFM micrographs suggest that young epidermis contains more integrin-α6+ basal cells (Fig. 5a). Quantitative analysis of the stratum basale revealed an age-dependent decline of integrin-α6+ basal cells from ≈7% in young (<30 y: 6.6 ± 0.7%) to ≈3% in middle-aged (30–60 y: 3.3 ± 0.2%) and aged epidermis (>60 y: 2.8 ± 0.2) (Fig. 5a, right panel). The marked stain of integrin-α6 along the basal surface of keratinocytes in young epidermis may result from the close interplay of this integrin with the basement membrane. Previous studies by immunoelectron microscopy have shown that integrin-α6 localize to hemidesmosomes, and thus may play a critical role in promoting the adhesion of epidermal stem and progenitor cells to the underlying basement membrane^32^. However, differences in the levels of this cell surface protein among basal keratinocytes are difficult to detect using immunohistochemistry^31^.

In excisional biopsies from abdominal skin, epidermal thickness was measured by computer-supported image analysis of histopathological sections. In hematoxylin–eosin-stained vertical cross-sections epidermal areas were measured from basal membrane to the stratum corneum (Fig. 5b, left panel). The analysis of the different age groups revealed that epidermal thickness was correlated to chronological donor age (Fig. 5b, right panel). Moreover, the age-related decline of epidermal thickness was associated with slight, but gradual decline of integrin-α6+ stem/precursor cells (Fig. 5b, right panel). Together, our findings suggest that the numerical and functional decline of epidermal stem/progenitor cells may contribute to diminished tissue homeostasis with age.

## Discussion

Classically, two types of skin aging are differentiated: intrinsic aging associated with photo-protected areas and extrinsic aging owing to UV exposure on photo-exposed areas. Extrinsic aging may superimpose to intrinsic skin aging, depending on intensity, duration and chronicity of UV exposure^33^. Here, we analyzed H2A.J expression in biopsies of photo-protected skin of healthy individuals to primarily study the intrinsic aging process. With advancing age, we observed significant accumulation of epidermal keratinocytes with H2A.J+ nuclei, likely reflecting non-dividing, senescent cells. Skin biopsies of middle-aged donors (30–60 y) revealed the greatest variability of H2A.J+ keratinocytes, suggesting that factors other than UV radiation may affect the aging process of these largely photo-protected abdominal skin biopsies. Genetic variation is expected to affect the intrinsic aging process, and other uncharacterized extrinsic factors may also contribute to variation. In the stratified squamous epithelium of the epidermis, basal stem/progenitor cell division yields subsets of daughter cells that undergo a coordinated program of cell cycle arrest, outward migration, and terminal differentiation. H2A.J positivity of keratinocytes in spinous and granular layers may therefore be related to physiological maturation processes, being associated with the transformation of keratinocytes from the actively dividing to the non-dividing state. Evaluation of specific epidermal layers revealed that the amount of aged H2A.J+ cells in stem/progenitor cell-containing basal layers, makes up the decisive difference between young and aged epidermis. With increasing age, the proliferative capacity of basal keratinocytes was reduced, leading to epidermal thinning. Our findings suggest that epidermal stem/progenitor cells are mostly retained during skin aging (no statistical difference in the number of integrin-α6+ basal cells between middle-aged and aged donors, Fig. 5a), but their age-dependent increased entry into the senescent state (increase of H2A.J+ cells in stratum basale and spinale from middle-aged donors, Fig. 1c), may lead to altered regenerative potential and functional decline with impaired epidermal homeostasis. Our findings are in line with previous studies in mouse models, demonstrating that epidermal stem cells are retained throughout life, despite significant age-associated changes in epidermal proliferation and dermal thickness^34,35^.

Classical senescence biomarkers such as SA-β-Gal activity, overexpression of cell cycle arrest proteins or lipofuscin detection have been used for the identification and quantification of senescent cells in human skin. Even if senescent cells share common biomarkers, various skin subpopulations can potentially develop specific senescent phenotypes, depending on cell-type, localization, and tissue context. Consequently, the use of multiple markers is mandatory to adequately identify the senescent state. The most commonly used senescence biomarker SA-β-Gal allows the detection of lysosomal enzyme activity by X-Gal hydrolysis. However, our SA-β-Gal staining performed on fresh skin samples under controlled pH conditions failed to reveal senescent cells in our human epidermis samples. This agrees with recent studies that call into question the validity of pH 6 β-Gal activity as a senescence biomarker, and suggests it may instead represent a non-specific marker of lysosomes^36^. Analyzing human skin biopsies by validated senescence markers, p16^Ink4a^ overexpression, and lipofuscin detection, revealed senescent cells in basal layers of aged epidermis samples. These different staining patterns may reflect that various senescence factors, and thus different molecular pathways, are involved in triggering and/or maintaining varying aspects of the senescent phenotype. Our ongoing in vitro studies indicate that H2A.J incorporation into the chromatin of senescent cells leads to structural modifications and is associated with secretion of growth factors, cytokines, and chemokines, collectively known as the SASP^23,25^. Epigenetic mechanisms modify chromatin structure and DNA accessibility to shape the gene expression of keratinocytes in different epidermal layers and allow dynamic and coordinated expression changes to finely balance keratinocyte self-renewal and differentiation processes. This balance between proliferation and differentiation is tightly regulated to ensure the maintenance of the stem cell population and to preserve epidermal homeostasis during life.

In the literature, there are inconsistent findings regarding potential age differences for the Ki67 expression in human skin^37–39^. In addition, quantitative and topographic expression levels of Ki67 may vary in human skin of different anatomical locations^40^. In our study, analyzing abdominal skin of healthy volunteers, the prevalence of Ki67+ cells in the epidermis decreased significantly with chronological age (Fig. 2a). For these investigations, skin samples of three donors per age-group were selected based on their average H2A.J expression level. This selection procedure may probably lead to smaller Ki67 variations, owing to the inverse H2A.J/ Ki67 correlation.

Owing to the lack of specific markers for keratinocyte stem cells, it was not possible to examine whether stem or progenitor cells in aged epidermis may express H2A.J. The age-dependent increase of H2A.J in keratinocytes of basal and suprabasal layers suggest a different coordinated role during epidermal differentiation. The inverse correlation between Ki67+ and H2A.J+ keratinocytes in basal layers may reflect the involvement of H2A.J in the cell cycle exit. However, even in young epidermis, we observed an intense H2A.J expression in postmitotic keratinocytes of granular layers, suggesting that H2A.J may promote their terminal differentiation program. Collectively, the increased H2A.J expression levels in all epidermal layers of aged skin may reflect reduced cell proliferation and enhanced differentiation. p16^Ink4a^ has a well-established role in mediating and maintaining cellular senescence during both replicative and stress-induced senescence processes. Previous studies have shown that p16^Ink4a^ accumulates during tissue aging and therefore is considered to be a robust senescence biomarker. Indeed, p16^Ink4a^ expression was undetectable in healthy, unstressed skin of young donors, but some p16^Ink4+^ basal keratinocytes were detected in aged epidermis. However, specific double-staining of p16^Ink4a^ and H2A.J for senescence detection could not be established in human skin to date. Our in vitro studies on human fibroblasts show that the H2A.J incorporation occurs gradually during senescence progression, but alter profoundly the chromatin structure in senescent cells and ultimately induces SASP^25^. Analyzing skin sections, we observed a wide range for the H2A.J staining intensity, with weakly to strongly positive keratinocytes in human epidermis (Supplementary Figure 4). This phenomenon may also reflect chromatin rearrangements during the physiological differentiation program of keratinocytes. Accordingly, different levels of H2A.J positivity in keratinocytes may comprise various stages of the differentiation process, from terminally differentiated to early or even deep senescent cells. Notably, even weakly H2A.J+ cells show no Ki67 expression (Supplementary Figure 4). Previous studies analyzing the expression of the senescence marker p16^INK4A^ in human skin have shown that melanocytes (identified by their Melan A expression) are the main senescent cell population in the epidermis epidermis^41,42^. Analyzing the H2A.J expression in Melan A+ cells in human skin sections, we observed consistently very strong H2A.J signal intensities in the nuclei of aged melanocytes (Supplementary Figure 4). Based on our findings, we presume that only the highly H2A.J+ cells (comprising only a few percent in aged epidermis) are in a senescent-like state. Future studies have to elucidate the precise role of H2A.J during senescence progression in human skin aging.

As telomeres progressively shorten through cell division, telomere length may be an indicator for chronological aging^15,43,44^. Despite substantial variation in average telomere lengths among same-age individuals (presumably as a consequence of variation in both genetic, lifestyle, and environmental factors), we noted an age-dependent telomere-length reduction up to the age of 60 years in blood lymphocytes and up to the age of 50 years in epidermal keratinocytes. Unexpectedly, some aged individuals (>60 y) had longer average telomere lengths, suggesting that the positive selection of healthy elderlies (suitable to overcome stressful surgery) may have biased our study. However, similar findings have been made in other population samples, e.g., in aged workers of the Mayak nuclear weapon production facility^29^ or in cancer patients after therapeutic irradiation^45^. These findings support the idea that telomere length is indicative of biological fitness of individuals and not primarily of chronological age^46^. Collectively, the pronounced variability of age-dependent telomere lengths, particularly in middle-aged individuals, suggests that telomere length and integrity are regulated at different levels during the complex aging process^47^. Previous studies have shown that telomerase is expressed in the human epidermis independent of age and that telomerase activity can potentially counteract telomere-length reduction with advancing chronological age^48^. Shortening or damage to telomeres and opening of the loop can induce an uncapped state that triggers the DDR resulting in cellular senescence^49,50^. Our quantitative analysis revealed that telomere-associated 53BP1-foci increased with chronological aging, but the age-dependent increase of H2A.J+ keratinocytes was clearly more pronounced, suggesting that H2A.J is a more sensitive biomarker for senescence identification in the human epidermis.

Communication between senescent cells and their microenvironment may play an important role in the pathophysiology of skin aging^5,51^. One major challenge for future studies is to investigate the underlying cause of accumulating senescent cells (intrinsic versus extrinsic stressors) and to explore the functional impact of senescent cells on epidermal homeostasis.

## Methods

### Human skin and blood samples

Skin and blood samples were collected from healthy volunteers of different ages (18–90 years, *n* = 53) with no severe underlying diseases. Excisional biopsies (≥5 mm) were obtained from sun-protected abdominal skin during surgical procedures. Peripheral blood was collected in sodium heparin-containing vacutainers and lymphocytes were isolated immediately by density gradient centrifugation (Percoll™, Merck, Darmstadt, Germany). Voluntary donors were divided into three age groups: young: <30 years (*n* = 12), middle-aged: 30–60 years (*n* = 20), and elderly: >60 years (*n* = 21). For detailed analysis of age-dependency, data were depicted in 10-year age groups (<30 y, 30–40 y, 40–50 y, 60–70 y, 70–80 y, >80 y). Protocol procedures were approved by the local ethics committee (“Ethikkommission der Ärztekammer des Saarlandes”) and all donors provided written and informed consent.

### Irradiation of cultured human skin samples

Skin explants were divided into sections and rinsed with PBS. Skin sections were incubated dermal side down on polyethylene membranes and epidermal side exposed to air in six-well plates (each well filled with 2 ml medium) at 37°C, under 5% CO_2_. The culture medium consisted of Dulbecco’s Modified Eagle Medium supplemented with 10% fetal calf serum, 10,000 units penicillin, 10 mg/ml streptomycin, and 200 mM/l glutamine. After air-medium interface cultivation for 12 hours, skin sections were exposed to IR with 10 Gy (6-MV photons, 2 Gy/min) using the linear accelerator Artiste™ (Siemens, Munich, Germany). 48 h after IR exposure, cultured epidermis sections were harvested for paraffin embedding and subsequent IFM analysis. Histopathological studies confirmed the viability and functionality of our skin organ culture method.

### Murine skin of H2A.J KO mice

TALEN (transcription activator-like effector nucleases)-mediated genome engineering was used to generate the H2A.J KO mouse model on the genetic background of C57Bl/6-N mice (Cyagen, Santa Clara, CA, US). At the beginning of the H2AFJ gene, a 7 bp deletion was introduced in the C57Bl/6-N genome of murine oocytes. This H2AFJ∆7 mutation created a frameshift with a premature stop codon at the start of the coding sequence; consequently, this gene disruption resulted in a truncated, non-functional protein product. The founder heterozygous mutants were back-crossed six times to C57BL/6-N mice (wild-type, WT, Janvier Laboratory, France). Homozygous H2A.J KO mice are viable and fertile and with no apparent anatomical abnormalities.

WT and KO mice were exposed to different doses with 6-MV photons at the linear accelerator (Artiste™, Siemens). At defined time-points after whole-body irradiation, animals were anesthetized by the intraperitoneal injection of a mixture of ketamine/xylazine, prior to intracardial perfusion and tissue collection for IFM analysis. These studies were approved by the Medical Sciences Animal Care and Use Committee of the University of Saarland.

### Immunofluorescence microscopy

Formalin-fixed skin tissues were embedded in paraffin and sectioned to 4 µm thickness. After dewaxing in xylene and rehydration in decreasing concentrations of alcohol, sections were boiled in citrate buffer and incubated with 2% goat serum (Carl Roth). Sections were incubated with primary antibodies (anti-H2A.J, 1:800, Active Motive, LaHulpe, Belgium, Cat. No. 61793; anti-53BP1, 1:500, Merck KGaA, Darmstadt, Germany, Cat. No. Mab3804; anti-Ki67, 1:500, Agilent, Santa Clara CA, USA, Cat. No. M7240; anti-Integrin-α6, 1:500, Abcam, Cambridge, UK, Cat. No. ab181551; anti-H2A, 1:500, Abcam, Cambridge, UK, Cat. No. ab177308; anti-H2AX, 1:500, Abcam, Cambridge, UK, Cat. No. ab20669; anti-γH2AX, 1:500, Bethyl Laboratories, Montgomery, TX, USA, and anti-Melan A, 1:200, Santa Cruz Biotechnology, Heidelberg, Germany, Cat. No. sc20032) followed by AlexaFluor-488 or AlexaFluor-568 secondary antibodies (1:400, Invitrogen Carlsbad CA, USA, Cat. No. A11034 and A11031). In addition, anti-H2A.J antibodies generated from mouse, rabbit, and guinea pig were applied as efficient investigation tools for various test requirements. Finally, sections were mounted in VECTAshield™ with 4′,6-diamidino-2-phenylindole (DAPI; Vector Laboratories, Burlingame, USA). For quantitative analysis ≥1000 epidermal cells were registered for each sample and H2A.J+ and Ki67+ cells were counted using Nikon E600 epifluorescent microscope (Nikon, Düsseldorf, Germany). To analyze the distribution of H2A.J in different epidermal layers, H2A.J+ cells were enumerated separately in stratum granulosum/spinosum/basale and the relative amounts of H2A.J+ cells per layer were depicted for the different age groups. 53BP1-foci were counted in all epidermal layers until at least 40 foci and/or 200 cells were registered for each skin sample. In double-labeled skin sections, correlations between 53BP1-foci and H2A.J expression were analyzed by additionally enumerating double-positive cells.

### Interphase quantitative fluorescence in situ hybridization

For relative telomere-length measurement in acetic acid/methanol fixed lymphocytes, IQ-FISH was applied with a peptide nucleic acid (PNA) telomere probe (Dako, Glostrup, Denmark) in combination with differentially colored centromere 2 PNA probe, as described^29,52^. Per individual ≈980 lymphocyte nuclei (range 440–3200) displaying telomere and two centromere signals were analyzed. Mean relative telomere lengths were expressed as arbitrary fluorescence ratio units of telomere and centromere FISH signal fluorescence intensity.

Relative telomere lengths in skin sections were determined using Cy3-labeled PNA-TTAGGG probes (DAKO) and tissue FISH to dewaxed paraffin sections, as described^53,54^. In brief, skin sections were pre-treated with 1 M NaSCN (Carl Roth, Karlsruhe, Germany) and Pepsin (4 mg/ml, pH2) and RNAse (10 mg/ml in PBS) digested. Sections were denatured and hybridized at 37°C ON, followed by washes in 0.025× SSC at 45°C. After embedding in antifade solution (Vectashield, Vector laboratories, Burlingame, CA, USA) automated image recording was performed with Metafer imaging system (MetaSystems, Altlussheim, Germany). Quantitative Telomere FISH signal analysis comprised ≈1420 epidermal keratinocyte nuclei (range 300–2500) per patient in interactively selected epidermis regions. Since collagen autofluorescence often compromised Cen2-FISH signals, relative telomere-length values were computed from Telo-FISH fluorescence intensities using Strataquest software (TissueGnostics, Vienna, Austria). Relative telomere lengths were expressed by arbitrary fluorescence units, as ratio of the entire area covered by telomere signals relative to DAPI-blue nuclei.

### Simultaneous IFM/FISH

After deparaffinization, rehydration, and antigen retrieval, tissue sections were incubated with PNA probe (800 ng/ml, Panagene Inc., Daejeon, Korea) diluted in hybridization buffer (for 5 min at 80°C, then for 2 h at 37°C). After hybridization, sections were washed, incubated with blocking buffer, and finally stained for 53BP1, as described^23^. To analyze the relationship between DNA damage foci and uncapped telomeres, potential colocalization of 53BP1-foci (IFM) and telomere signal (FISH) was quantified in epidermal cells by visual inspection. For every skin sample, at least 400 basal cells and/or 40 53BP1 + basal cells were analyzed for telomeric 53BP1-foci.

### Transmission electron microscopy

Skin samples were trimmed into 2 mm^3^ cubes and fixed overnight with paraformaldehyde and glutaraldehyde. Fixed samples were dehydrated using series of ethanol washes with increasing concentrations, infiltrated with LR Gold resin™ (EMS, Hatfield, USA), embedded in fresh resin with benzyl (EMS) and polymerized in the UV light cryo-chamber at 4°C. One-micron sections were taken to find the area of interest. Subsequently, ultrathin sections (≈70 nm) were cut on Ultracut UCT Leica™ with diamond knives (Diatome; Biel, Switzerland), placed on pioloform-coated nickel grids and incubated in Proteinase K, followed by blocking in glycine and blocking solution. After washing grids were floated on TelC-FITC (F1009, Discovery peptides, UK) followed by anti-FITC 10 nm gold-labeled secondary antibody to detect telomers. Subsequently, grids were further incubated with 53BP1 primary antibody, followed by 6 nm gold-labeled secondary antibody. Sections were rinsed and post-fixed with glutaraldehyde. Finally, sections were stained with uranyl acetate and examined by TEM (Tecnai Biotwin™, FEI Company/ThermoFisher Scientific Inc., Eindhoven, The Netherlands).

### Immunohistochemistry

After pre-fixation, skin samples were incubated in X-Gal staining solution (pH 6), followed by end-fixation overnight. Formalin-fixed, SA-β-Gal-stained skin were embedded in paraffin and sectioned at 7 µm thickness. After dewaxing in xylene and rehydration in decreasing ethanol concentrations, antigen retrieval was performed in citrate buffer, and sections were incubated with anti-H2A.J or anti-p16^Ink4a^ antibodies (1:500, Abcam, Cambridge, UK, Cat. no. ab54210) followed by biotin-labeled antibodies (Dako, Glostrup, Denmark). Staining was completed by incubation with 3,3′-diaminobenzidine and substrate chromogen. Finally, sections were counterstained with hematoxylin and mounted with Aqueous Mounting Medium (Dako, Glostrup, Denmark). Double-labeling for Lipofuscin was performed by incubation with a Sudan-Black-B solution, as described^55^.

In excisional biopsies from abdominal skin, epidermal thickness was measured using microscope imaging software NIS-Elements (Nikon, Düsseldorf, Germany). In hematoxylin–eosin-stained vertical cross-sections, five representative visual fields were captured with ×20 objective. In histological images, regions of interest (epidermal area from basal membrane to stratum corneum) were delineated, automatically scanned, and measured.

### Statistical analysis

Normally distributed data were presented as mean±SEM and potential differences between data groups were analyzed by *t* test. Integrin-α6 data were presented as box-plot showing median value, upper, and lower quantiles and analyzed using Mann–Whitney *U* test. All statistical analyses were performed by statistical software SPSS (SPSS Statistics25, IBM, Armonk, New York, USA). Statistical significance was presented as *p < 0.05, **p < 0.01, ***p < 0.001.

### Reporting summary

Further information on research design is available in the Nature Research Reporting Summary linked to this article.

## Supplementary information

Supplementary Figures

reporting summary

## Data Availability

The data that support the findings of this study are available from Department of Radiation Oncology, Saarland University Hospital, Homburg/Saar, Germany.

## Code availability

The data sets generated during and/or analyzed during the current study are available in the file repository, https://uks.sharefile.eu/d-sf24a1079cc40477f97c07a118d68b5e4.
