# Supplementary material for: Human skin aging is associated with increased expression of the histone variant H2A.J in the epidermis
Source: NPJ Aging Mech Dis. 2021 Apr 1;7:7. doi: 10.1038/s41514-021-00060-z (PMC8016850; doi:10.1038/s41514-021-00060-z)
Supplement: Supplementary file 1 — Supplementary Figures [file 41514_2021_60_MOESM1_ESM.pdf]

Supplementary Figure 1

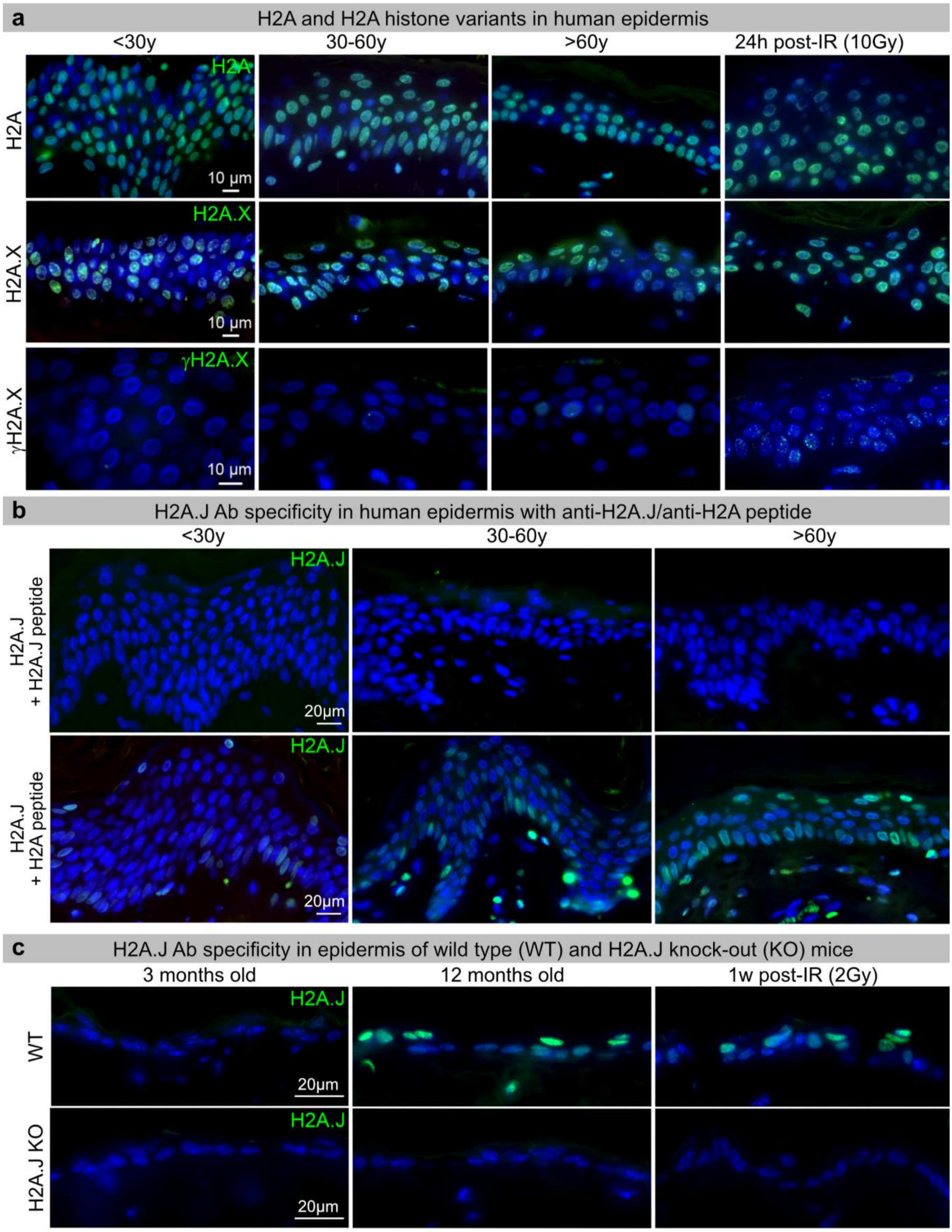

Validation of the H2A.J antibody binding specificity

(a) IFM micrographs of H2A, H2A.X and γH2A.X staining in young (<30y), middle-aged (30-60y), aged (>60y) and irradiated human epidermis (24h post-IR, 10Gy).

(b) IFM micrographs of H2A.J staining with anti-H2A.J and anti-H2A peptide in young (<30y), middle-aged (30-60y) and aged (>60y) human epidermis

(c) IFM micrographs of H2A.J staining in murine epidermis of 3-months old, 12-months old and irradiated (1w post-IR, 2Gy) of wild-type and H2A.J knock-out mice.

Supplementary Figure 2

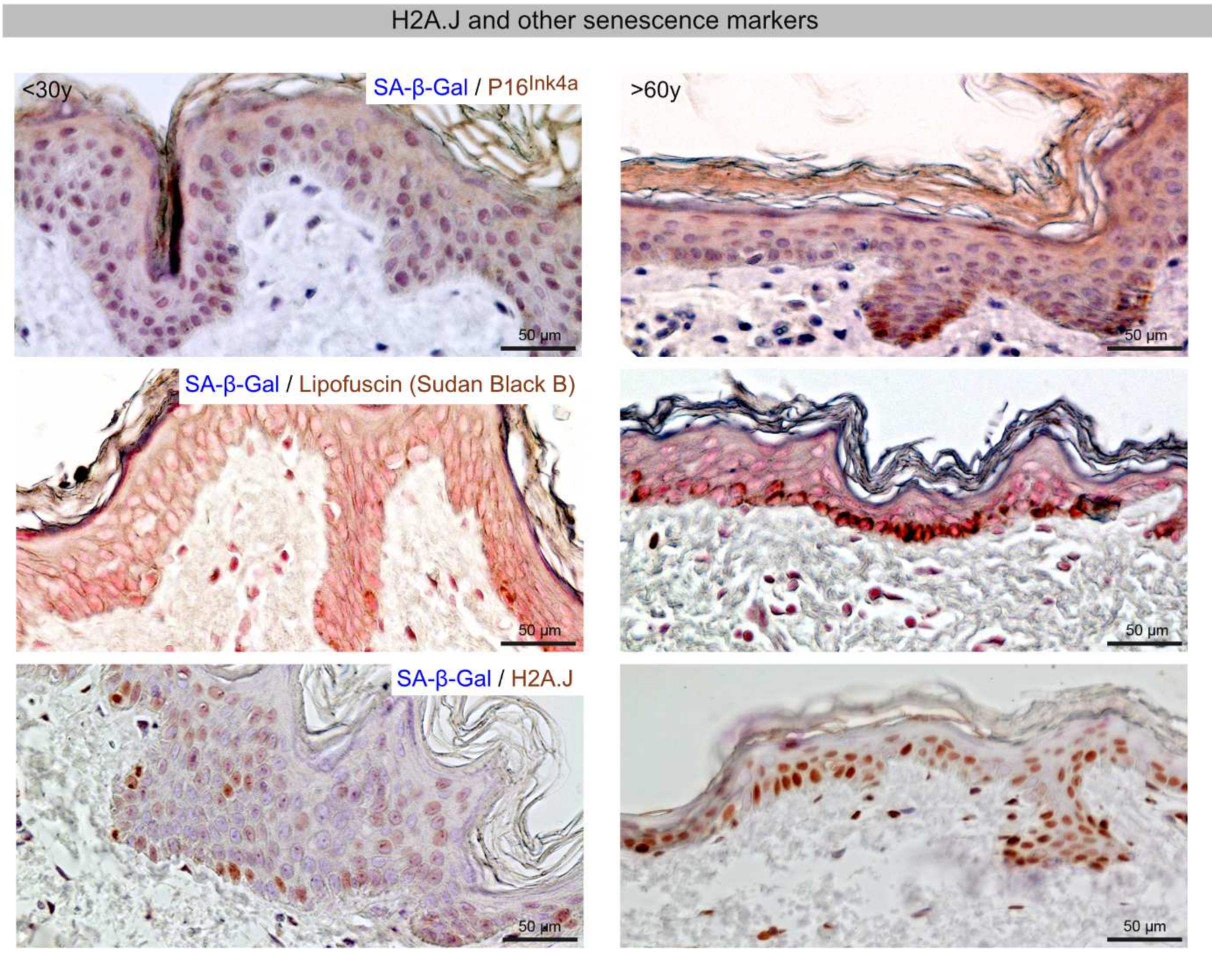

H2A.J and validated senescence markers

IHC micrographs of SA-β-Gal staining in combination with p16^{Ink4a}, lipofuscin and H2A.J in young (<30y) and aged (>60y) human epidermis.

Supplementary Figure 3

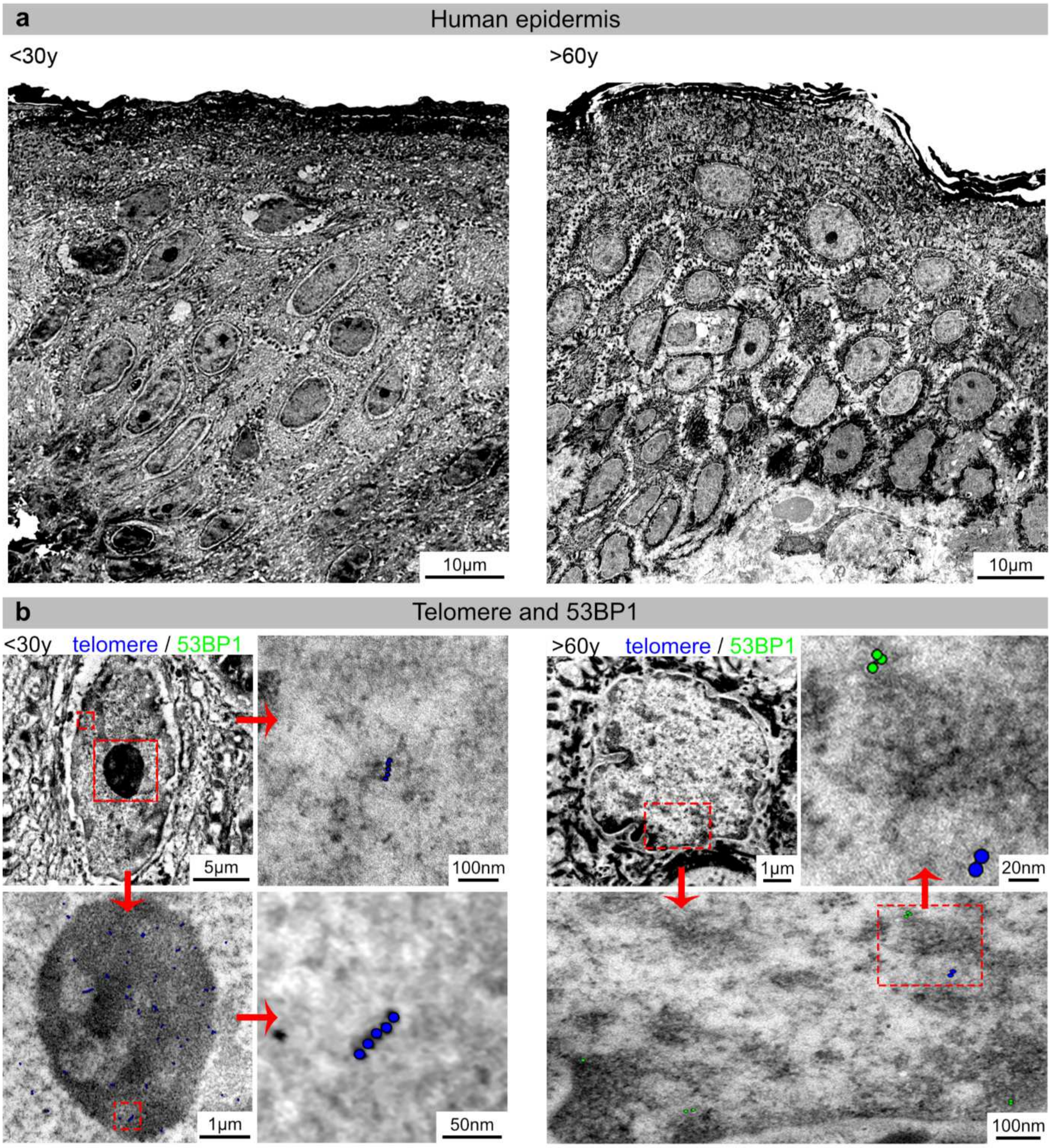

Telomer and 53BP1 in young and aged epidermis at the nanoscale level

(a) TEM micrographs show an overview of young and aged epidermis.

(b) In representative TEM images of young and aged epidermis, nuclei of keratinocytes revealed chains of telomere-specific gold-beads of different length, likely reflecting longer telomeres at young age (left panel, marked by blue dots), but shorter telomeres at advanced age (right panel). Significantly, telomere-specific sequences were always spatially distant from gold-labeled 53BP1 (superimposed with green dots).

Supplementary Figure 4

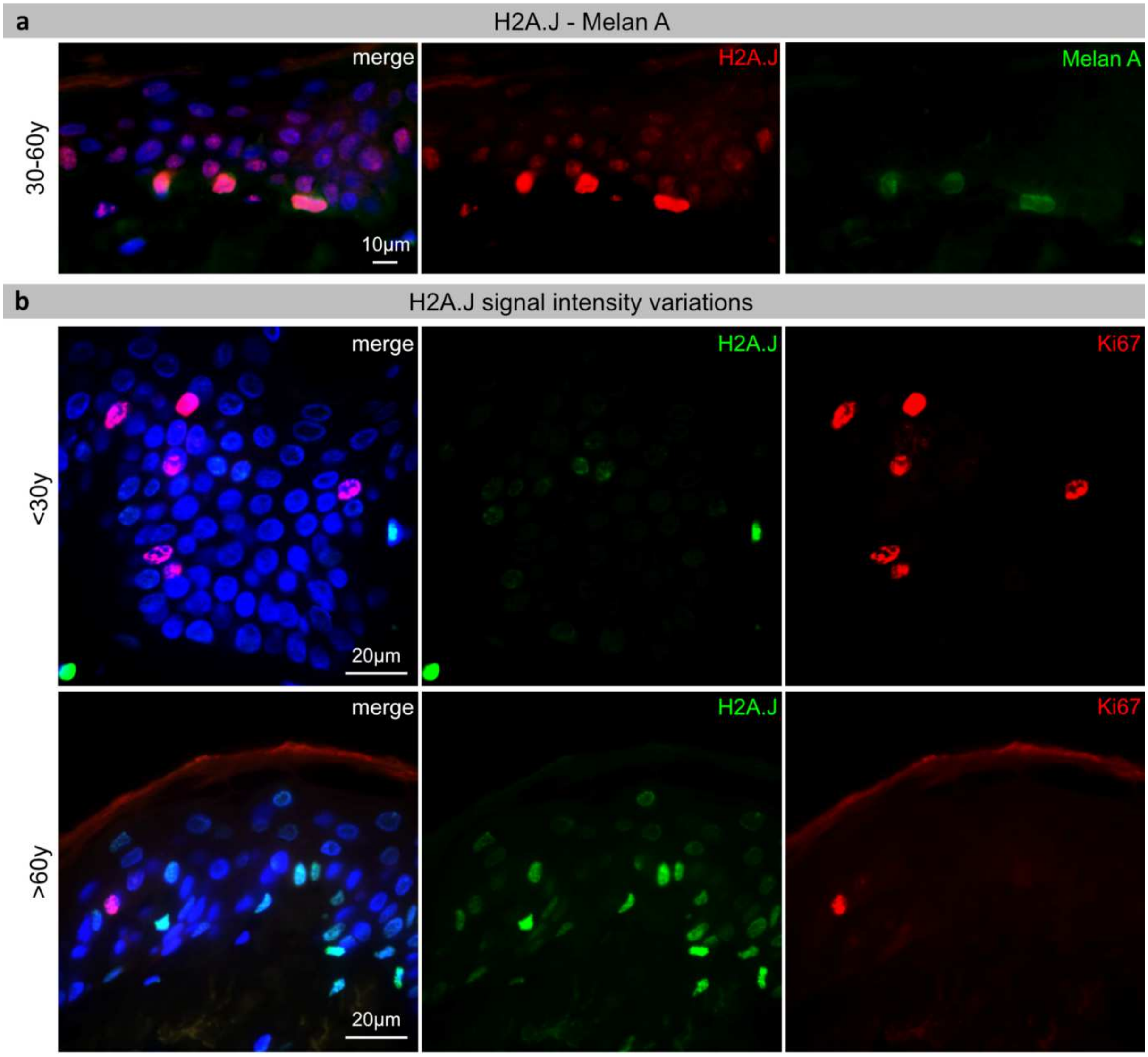

H2A.J expression of keratinocytes and melanocytes in human epidermis

(a) IFM micrographs of H2A.J/ Melan A double-staining in human epidermis of middle-aged donor (30-60y).

(b) IFM micrographs of H2A.J/ Ki67 double-staining in human epidermis of young (<30y) and aged (>60y) donors.
