# Supplementary material for: Human skin aging is associated with increased expression of the histone variant H2A.J in the epidermis
Source: NPJ Aging Mech Dis. 2021 Apr 1;7:7. doi: 10.1038/s41514-021-00060-z (PMC8016850; doi:10.1038/s41514-021-00060-z)
Supplement: Supplementary file 2 — reporting summary [file 41514_2021_60_MOESM2_ESM.pdf]

nature research

Corresponding author(s): Claudia E. Rübe

Last updated by author(s): 11th January 2021

Reporting Summary

Nature Research wishes to improve the reproducibility of the work that we publish. This form provides structure for consistency and transparency in reporting. For further information on Nature Research policies, see our Editorial Policies and the Editorial Policy Checklist.

Statistics

For all statistical analyses, confirm that the following items are present in the figure legend, table legend, main text, or Methods section.

n/a	Confirmed	
☐	☒	The exact sample size (*n*) for each experimental group/condition, given as a discrete number and unit of measurement
☐	☒	A statement on whether measurements were taken from distinct samples or whether the same sample was measured repeatedly
☐	☒	The statistical test(s) used AND whether they are one- or two-sided<br>*Only common tests should be described solely by name; describe more complex techniques in the Methods section.*
☒	☐	A description of all covariates tested
☐	☒	A description of any assumptions or corrections, such as tests of normality and adjustment for multiple comparisons
☐	☒	A full description of the statistical parameters including central tendency (e.g. means) or other basic estimates (e.g. regression coefficient) AND variation (e.g. standard deviation) or associated estimates of uncertainty (e.g. confidence intervals)
☐	☒	For null hypothesis testing, the test statistic (e.g. *F*, *t*, *r*) with confidence intervals, effect sizes, degrees of freedom and *P* value noted<br>*Give P values as exact values whenever suitable.*
☒	☐	For Bayesian analysis, information on the choice of priors and Markov chain Monte Carlo settings
☒	☐	For hierarchical and complex designs, identification of the appropriate level for tests and full reporting of outcomes
☒	☐	Estimates of effect sizes (e.g. Cohen's *d*, Pearson's *r*), indicating how they were calculated

Our web collection on statistics for biologists contains articles on many of the points above.

Software and code

Policy information about availability of computer code

Data collection	No software was used to for data collection.
Data analysis	Origin and SPSS were used for data analysis.

For manuscripts utilizing custom algorithms or software that are central to the research but not yet described in published literature, software must be made available to editors and reviewers. We strongly encourage code deposition in a community repository (e.g. GitHub). See the Nature Research guidelines for submitting code & software for further information.

Data

Policy information about availability of data

All manuscripts must include a data availability statement. This statement should provide the following information, where applicable:

- Accession codes, unique identifiers, or web links for publicly available datasets
- A list of figures that have associated raw data
- A description of any restrictions on data availability

The data that support the findings of this study are available from Saarland University Hospital, Homburg/Saar, Germany: https://uks.sharefile.eu/d-sf24a1079cc40477f97c07a118d68b5e4

Field-specific reporting

Life sciences study design

All studies must disclose on these points even when the disclosure is negative.

Sample size	Skin and blood samples were collected from healthy volunteers of different ages (18-90 years, n=53) with no severe underlying diseases. Excisional biopsies (≥5mm) were obtained from sun-protected abdominal skin during surgical procedures. Peripheral blood was collected in sodium heparin-containing vacutainers and lymphocytes were isolated immediately by density gradient centrifugation. Voluntary donors were divided into three age-groups: young: <30 years (<30y, n=12), middle-aged: 30-60 years (30-60y, n=20), and elderly: >60 years (>60y, n=21). For detailed analysis of age-dependency, data were depicted in 10-year age-groups (<30y, 30-40y, 40-50y, 60-70y, 70-80y, >80y). Protocol procedures were approved by the local ethics committee ("Ethikkommission der Ärztekammer des Saarlandes") and all donors provided written and informed consent.
Data exclusions	No data were excluded from the analysis.
Replication	2-3 replicates technical replicates were tested on all biologically samples. All attempts at replication were succesful.
Randomization	Samples were allocated into different age groups, to investigate the age dependency of the H2A.J expression.
Blinding	The investigators were blinded to group allocation during data collection.

Reporting for specific materials, systems and methods

We require information from authors about some types of materials, experimental systems and methods used in many studies. Here, indicate whether each material, system or method listed is relevant to your study. If you are not sure if a list item applies to your research, read the appropriate section before selecting a response.

Materials & experimental systems

n/a	Involved in the study	
☐	☒	Antibodies
☒	☐	Eukaryotic cell lines
☒	☐	Palaeontology and archaeology
☐	☒	Animals and other organisms
☐	☒	Human research participants
☒	☐	Clinical data
☒	☐	Dual use research of concern

Methods

n/a	Involved in the study	
☒	☐	ChIP-seq
☒	☐	Flow cytometry
☒	☐	MRI-based neuroimaging

Antibodies

Antibodies used	anti-H2A.J, Active Motive, LaHulpe, Belgium; anti-53BP1, Novus Biologicals, Abingdon, UK; anti-Ki67, anti-Integrin-α6, anti-H2A and anti-H2AX, Abcam, Berlin, Germany; anti-γH2AX; Bethyl Laboratories, Montgomery, TX, USA; anti-Melan A, Santa Cruz Biotechnology, Heidelberg, Germany)
Validation	Due to structural similarities between H2A.J, H2A and H2A.X, we aimed to exclude potential cross-reactivity of our H2A.J antibody, which would invalidate our results. However, double-staining for H2A.J with canonical H2A and histone variant H2A.X was technically not feasible, probably because their antigens are located on identical nucleosomal structures, impacting antibody binding by mutual steric hindrance. However, the single-staining patterns for canonical H2A and histone variant H2A.X in young, old and irradiated human epidermis are distinctly different from H2A.J, with no visible variations due to age or radiation exposure (Supp.1a). As expected, we observed only single γH2A.X-foci in non-irradiated epidermis of middle-aged and aged individuals, but multiple γH2A.X-foci in ex-vivo irradiated human skin (10Gy, 24h post-IR) (Supp.1a). Subsequently, the specifity of H2A.J staining in human epidermis was analyzed by combining the H2A.J antibody with peptides synthesized for the specific binding epitopes of H2A.J and H2A, respectively. In combination with H2A.J peptide, H2A.J staining in human epidermis was completely inhibited, but in combination with H2A peptide we observed the age-dependent H2A.J staining pattern (Supp.1b). These competition assays using the immunizing peptide to test for specificity, demonstrate that the H2A.J antibody binds specifically to the antigen against which it was raised. Testing antibody performance against genetically modified samples is another way to verify that an antibody recognizes a specific target. Accordingly, we analyzed murine epidermis of wild-type (WT) and H2A.J knock-out (KO) mice in relation to their age and exposed to ionizing radiation (IR), respectively. While young WT mice (3 months old) revealed nearly no H2A.J staining, older (12 months old) and irradiated WT mice (2Gy, 1w post-IR) revealed clearly higher H2A.J staining levels in their epidermis. In contrast, murine epidermis of KO mice (that do not express the target protein H2A.J) revealed absolutely no positive staining with the H2A.J antibody (Supp.1c). Collectively, these control experiments provide definitive proof for specificity of our H2A.J antibody.

Animals and other organisms

Policy information about studies involving animals; ARRIVE guidelines recommended for reporting animal research

Laboratory animals	Murine skin of H2A.J knock-out mice: A 7 bp deletion at the beginning of the H2AFJ gene was introduced by a TALEN-mediated DNA break in the C57Bl/6-N (Charles River) genome by Cyagen. This H2AFJΔ7 mutation created a frame shift with a premature stop codon

at the beginning of the H2AFJ coding sequence. The founder heterozygous mutation was then back-crossed 6 times to C57BL/6-N mice (wild-type, WT) provided by the Janvier Laboratory (France). Viable homozygous mutants were obtained after the second backcross. The homozygous H2A.J-knock-out (KO) mice are viable and fertile and their further phenotypic characterization will be described in a future publication. Whole-body irradiation (2Gy) was performed at the linear accelerator (Artiste™, Siemens), as described previously (Schmal, Isermann et al. 2019). 1 week after IR exposure animals were anesthetized intraperitoneally using ketamine (120µg per g body weight) and Rompun® (16µg per g body weight), prior to intracardial perfusion and tissue collection for IFM analysis. These studies were approved by the Medical Sciences Animal Care and Use Committee of the University of Saarland.

Wild animals	n/a
Field-collected samples	This study did not involve samples collected from the field.
Ethics oversight	These studies were approved by the Medical Sciences Animal Care and Use Committee of the University of Saarland (ID number 35/2016).

Note that full information on the approval of the study protocol must also be provided in the manuscript.

Human research participants

Policy information about studies involving human research participants

Population characteristics	Skin and blood samples were collected from healthy volunteers of different ages (18-90 years, n=53) with no severe underlying diseases. Peripheral blood was collected in sodium heparin-containing vacutainers and lymphocytes were isolated immediately by density gradient centrifugation (Percoll™, Merck, Darmstadt, Germany). Voluntary donors were divided into three age-groups: young: <30 years (<30y, n=12), middle-aged: 30-60 years (30-60y, n=20), and elderly: >60 years (>60y, n=21). For detailed analysis of age-dependency, data were depicted in 10-year age-groups (<30y, 30-40y, 40-50y, 60-70y, 70-80y, >80y).
Recruitment	Excisional biopsies (≥5mm) were obtained from sun-protected abdominal skin during surgical procedures.
Ethics oversight	Protocol procedures were approved by the local ethics committee (“Ethikkommission der Ärztekammer des Saarlandes”, ID number 226/16) and all donors provided written and informed consent.

Note that full information on the approval of the study protocol must also be provided in the manuscript.
